# Effect of iodinated contrast media on renal perfusion: A randomized comparison study in pigs using quantitative contrast-enhanced ultrasound (CEUS)

**DOI:** 10.1038/s41598-017-13253-y

**Published:** 2017-10-13

**Authors:** Philipp Lamby, Friedrich Jung, Stefanie Graf, Lotte Schellenberg, Johannes Falter, Natascha Platz-da-Silva, Stephan Schreml, Lukas Prantl, Ralf P. Franke, Ernst M. Jung

**Affiliations:** 1 Department of Plastic and Reconstructive Surgery, University Hospital Regensburg, Franz-Josef-Strauss Allee 11, 93053 Regensburg, Germany; 2 Institute of Biomaterial Science and Berlin-Brandenburg Centre for Regenerative Therapies, Helmholtz-Zentrum Geesthacht, Kantstrasse 55, 14513 Teltow, Germany; 3 Department of Anesthesiology, University of Regensburg, Franz-Josef-Strauss Allee 11, 93053 Regensburg, Germany; 4 Department of Radiology, University Hospital Regensburg, Franz-Josef-Strauss Allee 11, 93053 Regensburg, Germany; 5 Department of Dermatology, University Hospital Regensburg, Franz-Josef-Strauss Allee 11, 93053 Regensburg, Germany; 6 Central Institute for Biomedical Engineering, Department of Biomaterials, University of Ulm, Albert-Einstein-Allee 47, 89081 Ulm, Germany

## Abstract

The administration of iodinated contrast media (CM) can cause microcirculatory disorder leading to acute renal dysfunction. In a prospective, randomized investigation two CM (Iodixanol vs Iopromide) were compared in 16 pigs. Each animal received 10 intra-aortal injections (5 ml Iodixanol or 4.32 ml Iopromide). Microcirculation was assessed using contrast-enhanced ultrasound (CEUS) directly on the kidney surface using time-to-peak (TTP) and blood-volume-analysis. Macroscopic observations were documented. Post mortem residual CM distribution in the kidneys was detected using X-ray. TTP was significantly prolonged over the descending vasa recta of the Iopromide group. This coincided with a visible marble-like pattern on the kidney surface occurring in 30 out of 80 Iopromide-injections but in 4 out of 80 Iodixanol-injections (p = 0.007). The blood volume over the entire kidney did not change after Iodixanol-application, but decreased by about 6.1% after Iopromide-application. The regional blood volume in the renal cortex showed a tendency to decrease by about 13.5% (p = 0.094) after Iodixanol-application, and clearly decreased by about 31.7% (p = 0.022) after Iopromide-application. The study revealed a consistent influence of repeated injections of two different CM on the kidney perfusion using three different imaging methods (CEUS analysis, macroscopic observation and X-ray analysis).

## Introduction

It is widely accepted that intra-arterial administration of iodinated contrast medium (CM) can cause renal dysfunction. 30% of acute kidney insufficiencies^1^ are linked to pharmacological agents and 70% of these cases are assumed to be dose-dependent^2^. Pharmacologically-induced damage has been described in the vascular, tubular and interstitial systems of the kidney^3,4^. The higher incidence of renal dysfunction after repeated intra-arterial administration of CM is regarded as an indicator that various CM can be responsible for kidney damage^5,6^.

Contrast media-induced nephropathy (CIN) is a life-threatening complication of radiological interventions using iodinated CM^7^. Most cases of CIN seem to be reversible and are associated with a mild and transient impairment of renal function^8^.

Haemodynamic alterations, vasoconstriction of renal vessels and/or damage of tubular cells have all been reported as possible mechanisms by which iodinated CM could bring about CIN^1,9^. The retention of CM in kidney tissues over extended periods of time is also a point of concern, owing to the associated increase in duration of exposure to a potentially cytotoxic agent. On the other hand, *in vitro* studies have revealed that CM can lead to rapid disorders in the morphology of blood cells, depending on the type of CM applied: discocytes became echinocytes^10–12^ associated with a drastic change in the submembraneous skeleton into a box-like structure^13^ after incubation of erythrocytes in an Iopromide/plasma mixture. Such changes in erythrocyte morphology and subcellular structure are associated with a rigidification of erythrocytes^14^.

Contrast-enhanced ultrasound (CEUS) is widely applied to monitor and document the perfusion of the macro- and microcirculation^15–22^. CM is reported to cause disorders of the microcirculation in the kidneys of rats and mice^23,24^. However, in the unilobar kidneys of these animals, the urine is clearly more concentrated^25^, and the anatomical structure does not allow the comparison of results observed in these animals with those from studies in pigs or humans^26^. Therefore, the hypothesis that iodinated CM is able to induce a microcirculatory renal disorder was investigated in pigs following repeated supra-renal intra-aortal injections of CM.

## Results

### Measurement of kidney perfusion using contrast-enhanced ultrasound (CEUS): TTP-Analyses

**Figure 1:**
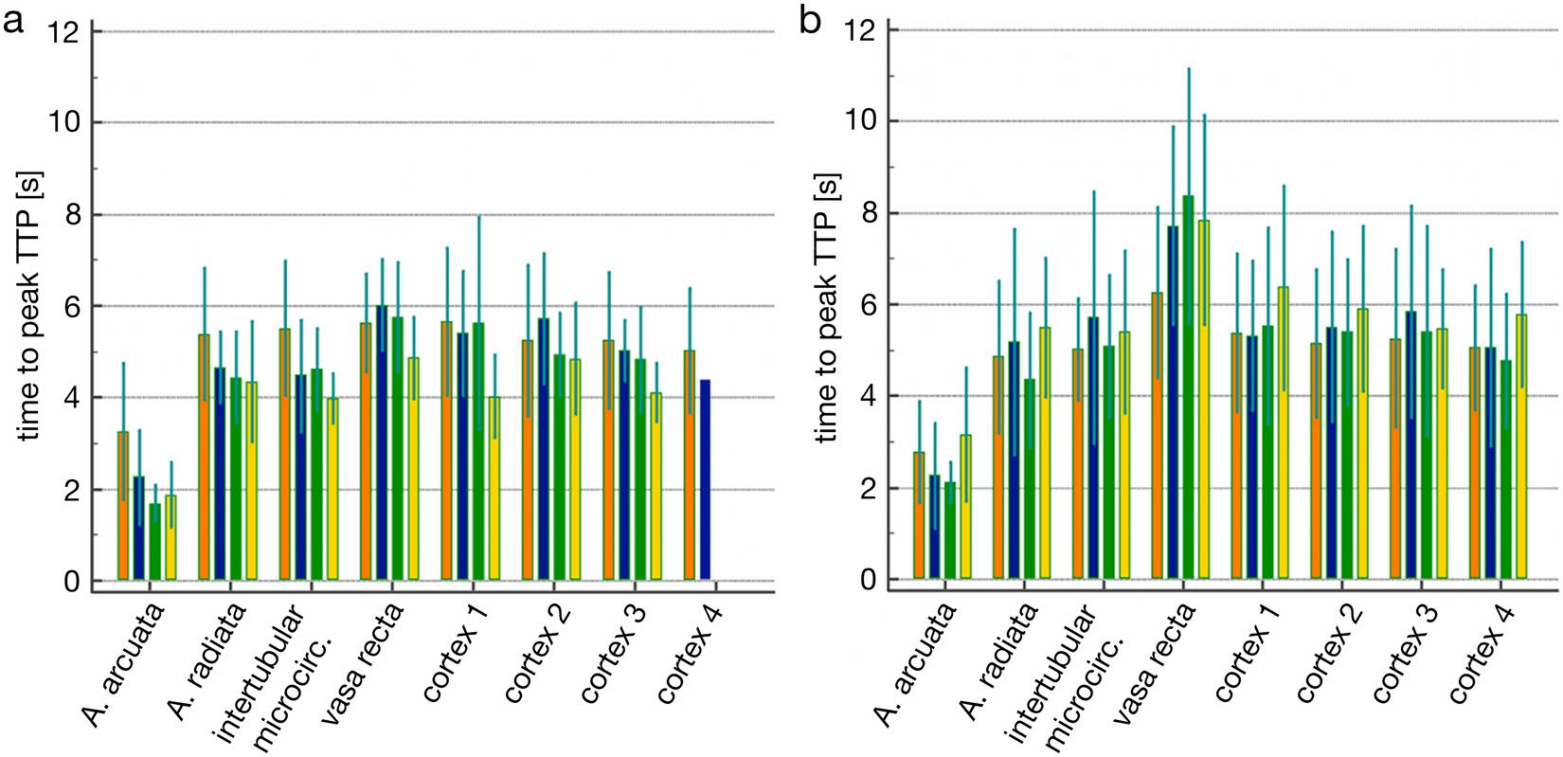
Comparison of the periods of time to reach the mean maximum intensity (TTP) of US contrast medium in different regional kidney blood vessels. Measured in the animal group receiving Iodixanol (**a**) and the group receiving Iopromide (**b**) at baseline (orange), after the first bolus (blue), the fifth bolus (green) and the tenth bolus (yellow).

The time to reach maximum signal intensity (Time-to-Peak/TTP) in the group of animals receiving Iopromide was greater than in the group receiving Iodixanol. There was only a slight difference in the peritubular capillary netnetwork, but quite a pronounced difference was observed in the descending vasa recta (Fig. 1).

There was a clear tendency towards prolongation of TTP in all observed regions of the kidney in the Iopromide-treated group, especially after injection of the tenth CM bolus. The time course of perfusion in the kidney regions observed after application of CM did not differ between the two groups (Greenhouse-Geysser-Test, CM × Injection: p = 0.232). The post-hoc analysis revealed, however, that regional perfusion in the Iopromide-group tended to be lower than in the Iodixanol-group after the tenth CM bolus (Scheffé test: p = 0.058). This tendency was also apparent when the mean TTP-values in the aa. corticalis radiatae were compared.

**Figure 2:**
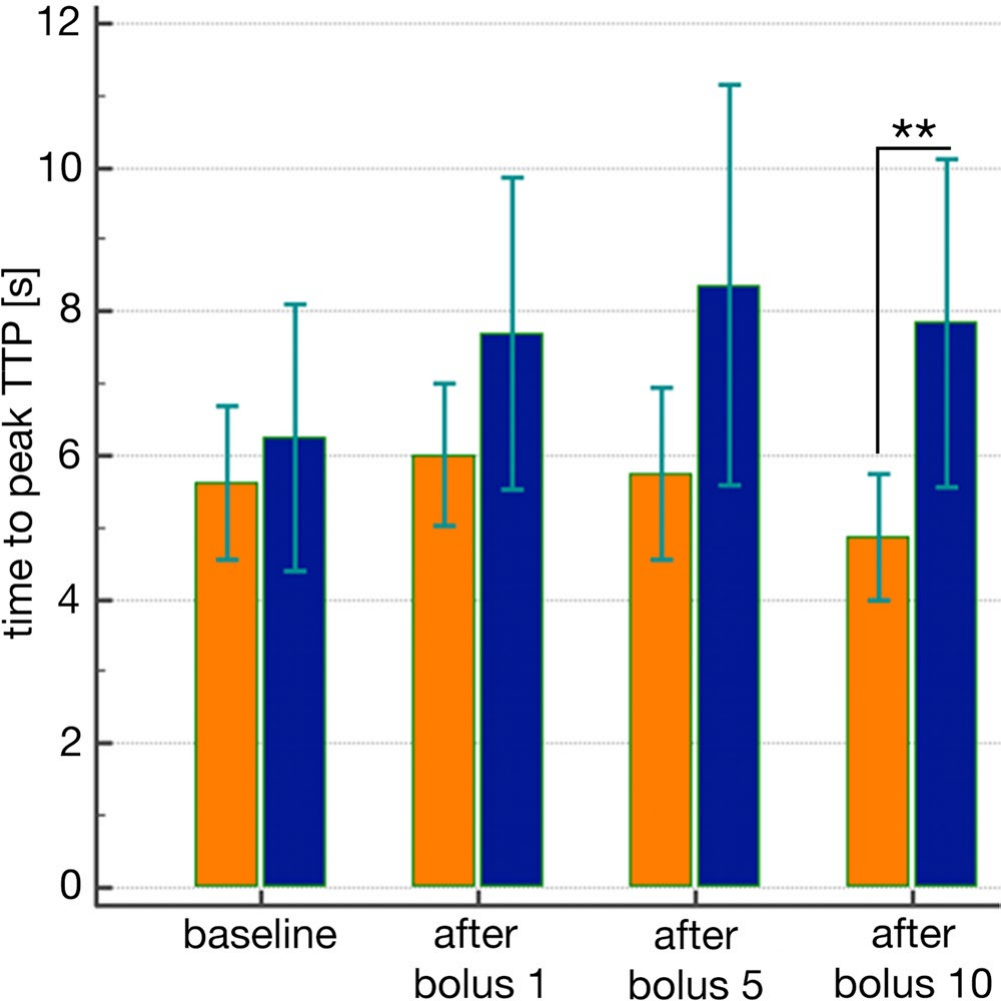
Comparison of the periods of time to reach the mean maximum intensity (TTP) of US-contrast medium. In the descending vasa recta in the animal group receiving Iodixanol (red column) and the group receiving Iopromide (blue columns) before CM application (baseline) and after the first, fifth and tenth CM bolus (mean values ± standard error, n = 8).

The situation with respect to TTP values was more pronounced in the peritubular capillary network. TTP-values in the Iopromide-group were significantly prolonged after the tenth CM bolus (Fig. 2). The post-hoc analysis showed that the mean times to peak-intensity were significantly greater and thus the regional blood flow lower in the Iopromide-group compared to the Iodixanol-group after the tenth CM bolus (Scheffé test: p < 0.001).

The time to peak-intensity values assessed in the descending vasa recta (Fig. 2) differed even more strongly between the two groups (p = 0.027) and were the clearest differences seen overall in this part of the examination. The post-hoc analysis demonstrated that kidney perfusion after the tenth CM bolus was significantly lower in the Iopromide-group compared to the Iodixanol-group (Scheffé test: p = 0.012).

The prolongation of TTP-values in the Iopromide-group was also evident in the surface-near cortical microcirculation.

### Haemodynamics

Heart rates were stable across groups throughout the entire examination period (CM × time p = 0.188), whereas mean systolic and diastolic blood pressures significantly increased over time by 19% in both treatment groups (p < 0.001). No differences in blood pressure were observed between groups (systolic: p = 0.682 and diastolic: p = 0.670).

Table 1 shows regional renal blood volumes of both groups before CM application and after injection of ten CM boli into the supra-renal aorta. While the blood volume of the whole kidney did not change after 10 Iodixanol boli, it decreased by about 6.1% after 10 Iopromide boli. In the surface-near region of the renal cortex, the regional blood volume showed a tendency to decrease by approximately 13.5% (p = 0.094) after injection of Iodixanol and decreased significantly by approximately 31.7% (p = 0.022) after injection of Iopromide.

**Table 1:** Regional blood volume in the kidneys before and after the tenth bolus of Iodixanol or Iopromide respectively (Qontrast analysis of CEUS database).

	Iodixanol			Iopromide		
	base line	after 10^th^ injection	p-value	base line	after 10^th^ injection	p-value
entire kidney	620 ± 499	627 ± 478	0.7571	624 ± 309	586 ± 277	0.3076
surface-near cortex	599 ± 541	518 ± 541	0.0940	460 ± 162	314 ± 83	0.0222

### CEUS database assessment of blood vessel diameter

**Figure 3:**
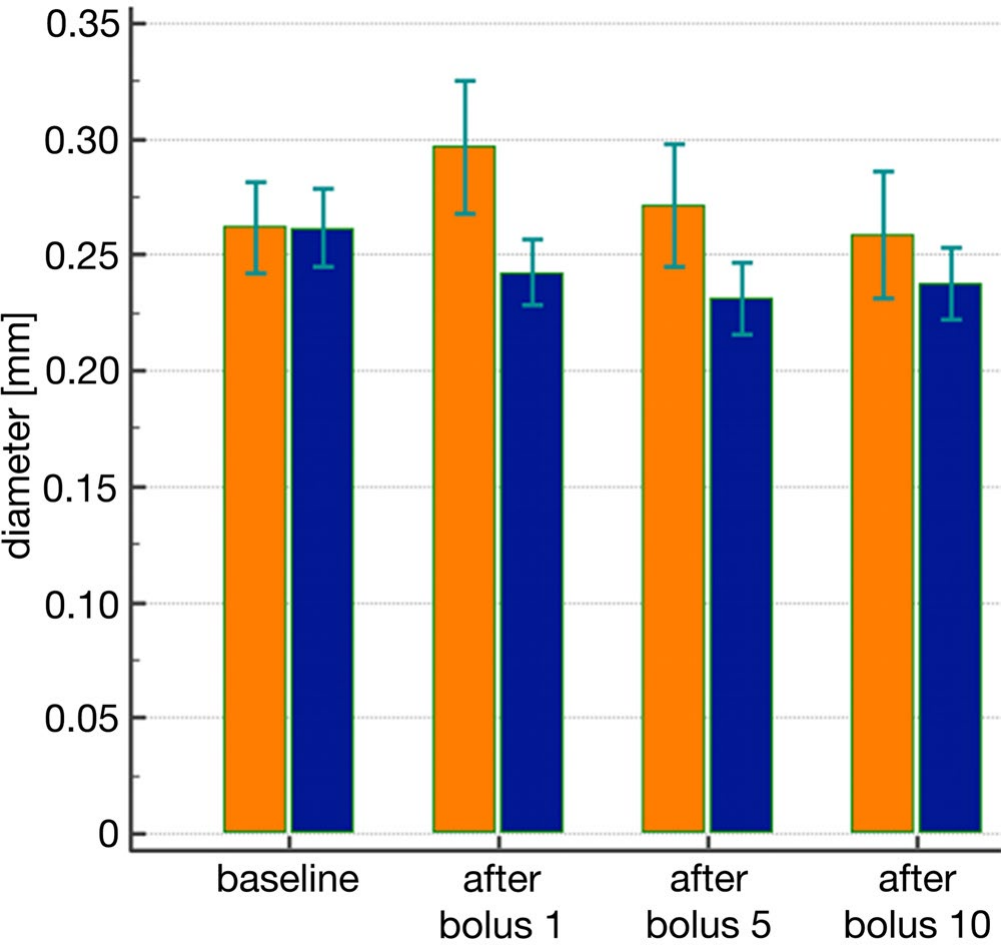
Mean diameter of Aa. arcuatae. Before CM application (baseline) and after application of the first, fifth and tenth CM bolus followed by US contrast-medium in the Iodixanol (orange) and Iopromide (blue) treatment groups (Sonovue™, Bracco, Konstanz, Germany).

Figure 3 displays the mean diameter of aa. arcuatae after application of the first, fifth and tenth CM bolus, followed by US-contrast-medium. Three diameter measurements of different arcuate arteries were taken per time point.

While there was a slight vasodilation, which returned to baseline values after Iodixanol application, there was a minor vasoconstriction after Iopromide application, which did not return to baseline values. The analysis of variance did not show any significant differences, however.

### Intravital marbling of the renal cortex following CM applications

During the examination, each injection of contrast medium was followed by a careful inspection of the kidney surface, which was documented photographically. In some cases, a reversible marble-like pattern appeared on parts of the surface of the kidney, especially, but not only, after application of Iopromide. The observed marbling varied in distribution and intensity (Table 2). Even in the case of distinct marbling, the effect practically disappeared within three minutes.

**Table 2:** Frequency of marbling of the kidney surface in both groups.

Animal	Iodixanol	Animal	Iopromide
1	none	9	after injection 1, 2, 3, 9, 10
2	none	10	after injection 6, 8, 9
3	none	11	after injection 1, 2, 3, 4, 5, 6, 7, 8, 9
4	none	12	after injection 2, 3, 5, 6, 8
5	slight, after injection 6	13	strong, after injection 8
6	none	14	after injection 2, 3, 5, 6
7	after injection 4, 6, 7	15	none
8	none	16	after injection 2, 5, 7, 8

4 out of 80 injections of Iodixanol (in 2/8 animals) were followed by marbling. 30 out of 80 injections of Iopromide (in 7/8 animals) were followed by marbling. The observed marble-like surface pattern appeared significantly more often after Iopromide application (Mann-Whitney U-test: p = 0.007).

The first clear differences in the time to peak-intensity of US contrast medium appeared in the a. arcuata after the tenth CM bolus, showing a slightly delayed inflow after Iopromide injection. This delay increased further in the Aa. corticalis radiatae, and the difference became significant in the peritubular capillary network. The time to peak-intensity after Iopromide injection was 27.3% longer than after Iodixanol injection. The greatest differences appeared in the descending vasa recta. Here the time to peak-intensity after Iopromide injection was 38% longer than after Iodixanol injection.

**Figure 4:**
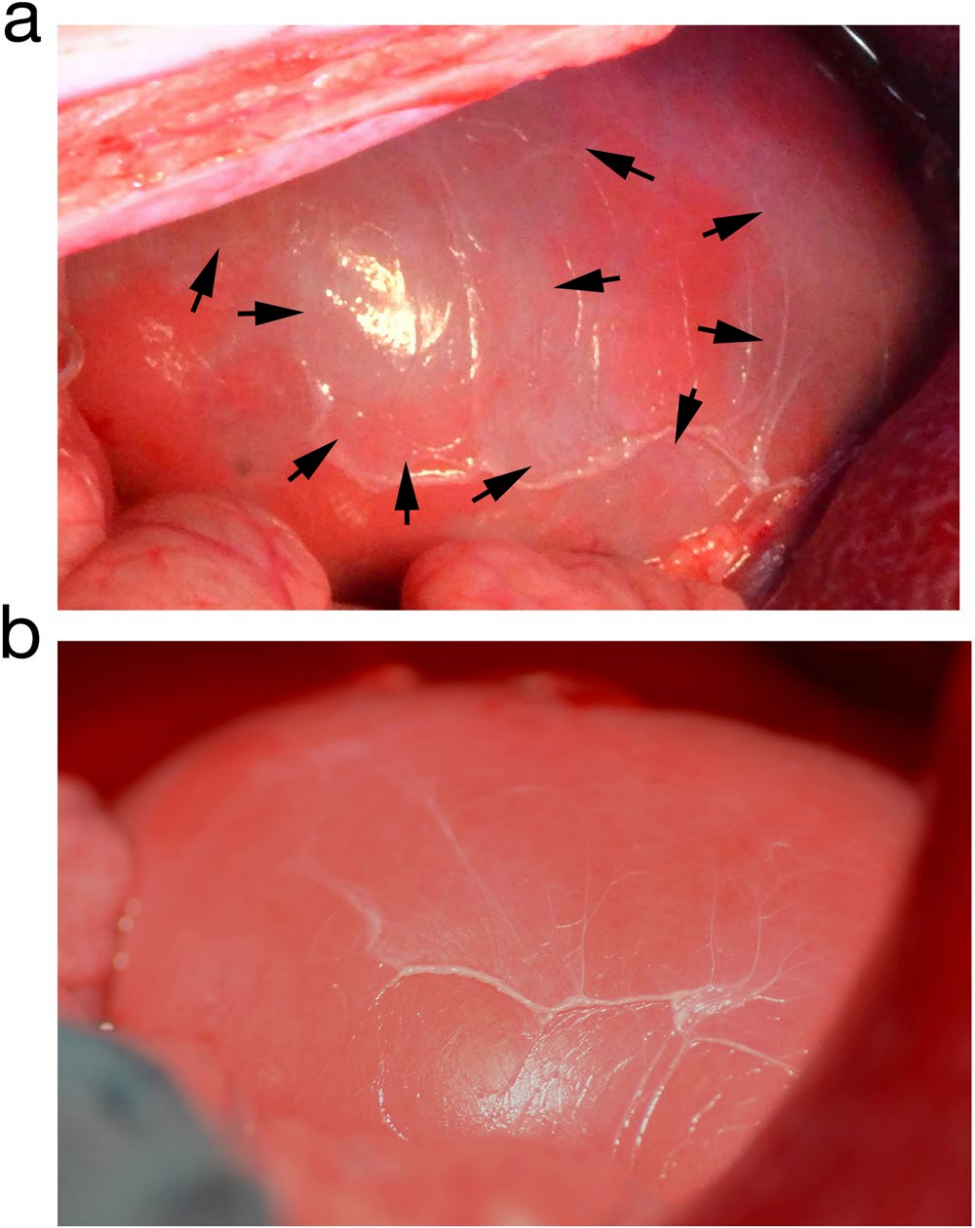
Photographs of the kidney. (**a**) Marbled kidney surface directly after the fifth Iopromide bolus. (**b**) Same kidney as shown in (**a**) surface marbling disappeared about 3 minutes after Iopromide-injection. At this time, the kidney appeared homogeneously pink again, the same shade of colour that was evident at the beginning of the examination.

Figure 4a shows an example of the kidney surface after the fifth injection of Iopromide. Clearly-demarcated grey-blue areas were observed, generating a marble-like pattern on the surface of the kidney (Fig. 4a).

### Radiography

**Figure 5:**
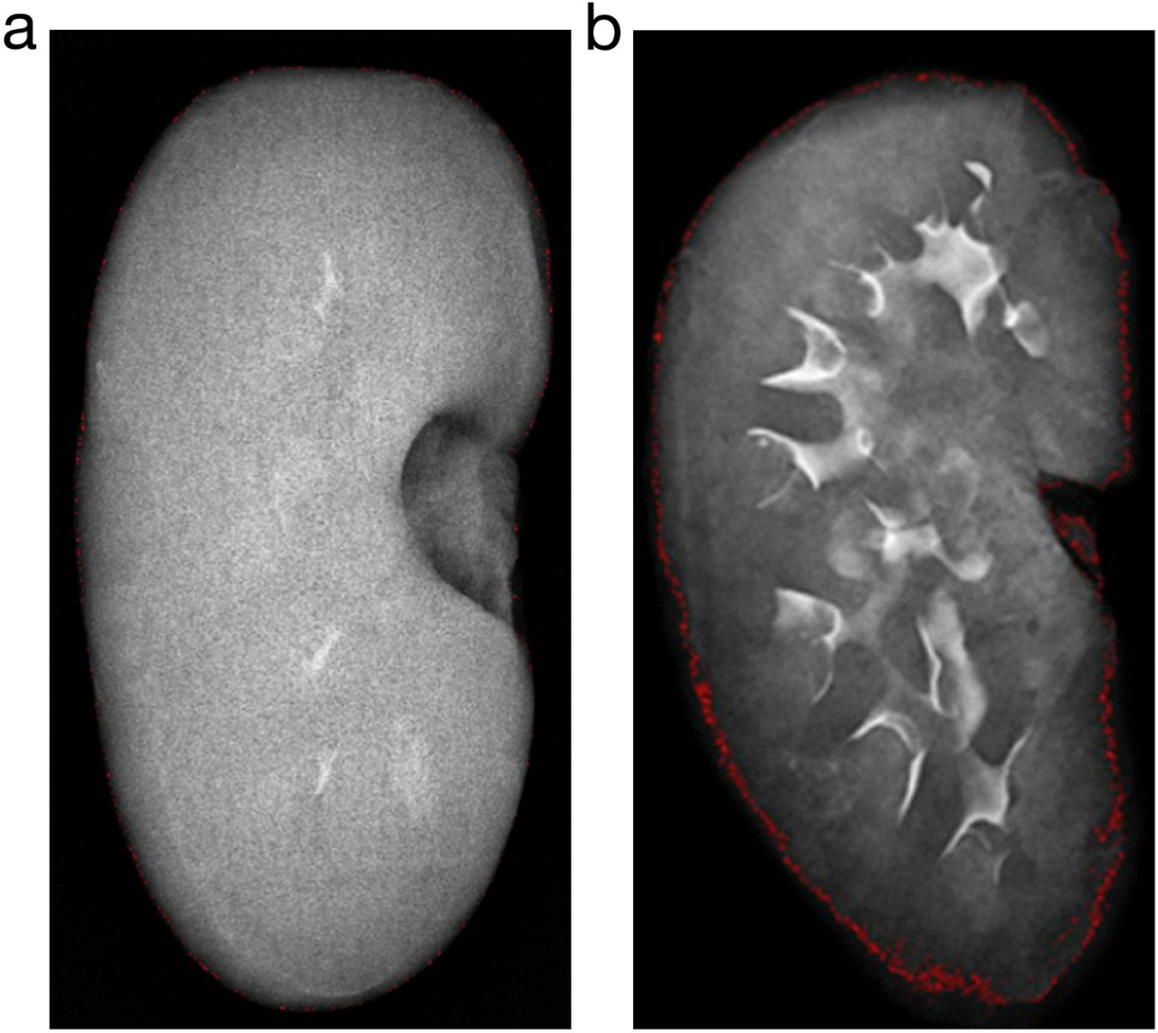
Radiography of kidneys. Explanted pig kidneys from the Iopromide-group (left) and the Iodixanol-group (right).

The red-dotted lines around the edge of the kidney are the result of a bit map evaluation and are composed of pixels with the lowest grey values measured within the X-ray image, marking the kidney border and indicating the transition from inside the kidney to the black background of the kidney X-ray (Fig. 5). There were regions exhibiting an essential disorder of the microcirculation and even maldistribution. The typical finding was low contrast in the renal cortex, which often coincided with the marbling effect observed on the kidney surface, primarily after application of Iopromide.

## Discussion

### Did the surgical procedure exert a major influence on the results of this study?

Heart rate remained constant during the examination period and did not differ between groups, while both systolic and diastolic blood pressures increased by about 19%.

It can be assumed that the rise in blood pressure in the kidney arteries resulted from the increase in systemic blood pressure measured in the aorta. Despite this increase in blood pressure, there was a significant prolongation in the time-to-peak-intensity in kidney arteries, coinciding with a decrease in blood velocity after application of Iopromide. The post-hoc analysis revealed that the time to peak-intensity after the tenth CM injection was significantly prolonged in the Iopromide group compared to the Iodixanol group (p = 0.027).

### Did the microcirculation in between contribute to the retardation of blood flow?

Treitl *et al*.^27^ have shown that repeated injections of Iodixanol do not induce an increase in the Renal Resistive Index (RRI).

All of the perfusion paths in the kidney (superficial, midcortical and iuxtamedullar) suffered from a retardation of perfusion after Iopromide injection, which became significantly different after the tenth CM bolus. In the surface-near cortical microcirculation, TTP after Iopromide injection was significantly delayed compared to TTP after Iodixanol. In two Regions of Interest (ROI) there was a trend towards retardation in one ROI, and no difference in the other ROI.

### Which mechanisms can be attributed to the retardation of blood flow?

The assessment of arterial diameters revealed a slight vasoconstriction after Iopromide injection, but a minor vasodilation after Iodixanol. Vasoconstriction, evidently, is not a general mode of action of CM because vasodilation also occurred with one type of CM. According to the Hagen – Poiseuille equation, small differences in vascular diameter would exert an influence on haemodynamics, since blood flow is proportional to the fourth power of the vascular diameter^28^. Therefore, even small changes in vascular diameter can be assumed to have a noticeable effect.

The marbling of the kidney surface points to local disorders in surface-near microvessels, which supply superficial nephrons, characterised by the highest oxygen demand as shown in pigs^29^. This local disorder of the microcirculation seems to be a very critical event. The macroscopically visible marble-like pattern was reversible in every case and disappeared after 3 minutes. While only 4 out of 80 Iodixanol injections were followed by surface marbling, significantly more, 30 out of 80, Iopromide injections led to this effect (p = 0.007). The marbling could be due to oxygen depletion combined with a change in colour of the haemoglobin in erythrocytes and/or a consequence of pure plasma flow in capillaries due to maldistribution.

The CEUS assessment in different kidney regions and the observed marbling effect did not only indicate a microperfusion disorder, but also a redistribution of blood from the superficial cortex to deeper regions of the underlying renal cortex and medulla. This was clearly shown and documented here for the first time. This seems to be of critical importance since the energy supply of nephrons in the superficial cortex strongly depends on oxygen transfer, whereas deeper layers are less or not at all dependent on oxygen.

In order to further document and quantify the redistribution of blood from the superficial cortex to deeper kidney layers after Iopromide injection, the CEUS database was analysed using Qontrast. In addition, the kidneys were X-rayed directly after explantation. The Qontrast analysis, based on the presence of US contrast medium, clearly showed that there was practically no blood redistribution after application of Iodixanol, although a marbling effect was visible in 2 out of 8 kidneys. There was a remarkable redistribution of blood after application of Iopromide, coinciding with surface marbling in 7 out of 8 kidneys. The X-ray images, based on the presence of CM, provide further evidence that a lower perfusion of the peripheral kidney zone resulted after application of Iopromide.

The unexpected marbling effect of kidney surfaces reveals an important fact; that marbling is reversible and can disappear within three minutes.

Although renal and intrarenal anatomy in the pig cannot be completely transposed to humans^30^, the similarities in size, physiology and organ development have established the pig as the gold-standard in renal transplantation research^26^ and as a suitable model for research into human diseases^31^. One limitation of this study relates to the use of young, healthy pigs, because CIN often occurs in multi-morbid patients of advanced age.

## Conclusion

A transient microcirculatory renal disorder that is dependent upon the type of the applied iodinated CM has been consistently demonstrated by three different imaging methods (CEUS analysis, macroscopic observation and X-ray imaging). This disorder clearly increased with the cumulative dosage of the CM applied, becoming significant after the 10th injection.

## Materials and Methods

### Study design

The study was performed as a prospective, randomized examination to compare the effects of two different CM (Iodixanol vs Iopromide) in pigs (n = 16). Animals in group I (n = 8) were randomised to Iodixanol, group II (n = 8) received Iopromide. To simulate the clinical setting, each animal received a total of 10 injections separated at five minute intervals via the suprarenal aorta, either with 5 ml Iodixanol per injection or with 4.32 ml Iopromide per injection, at a rate of 10 ml/sec^27^, so that both groups received equal amounts of iodine. Each animal received a total of 500 ml NaCl throughout the entire examination, which also mimics the clinical procedure.

The animals were placed on their backs with a slight hyper-extension of the lumbar spinal column. After sterile lavage and coverage of the non-operated regions, surgical access was gained through median laparotomy. Taking care not to endanger the intestine, the rectus sheath and the peritoneum were severed. Then the small intestine and the colon were dislocated outside the body. Trans-peritoneal access was gained and the v. cava, aorta, renal blood vessels and the ureter of the right kidney were identified. Following the ureter cranially, the renal hilus was identified and the kidney was released from the surrounding fatty tissue and the fibrous capsule. Then a catheter was inserted into the abdominal aorta from caudal to cranial aspect, with the tip lying 2–3 cm above the renal arteries for the injection of contrast medium.

Treitl *et al*. have shown that different iodinated contrast media (CM) can induce a more or less increase of the renal resistance index (RRI)^27^. The resistance of the microvasculature could play a decisive role in this increase in RRI. Since CEUS is able to assess the blood flow in capillaries, we selected CEUS as the most important diagnostic tool. In addition, because CM may remain in kidney tissues for extended periods of time, we chose different methods of radiography (mammography and CT) to show a possible accumulation and regionalization of CM in kidney regions directly after kidney explantation. X-ray imaging became more important after the marbling-effect of kidneys had been observed in the first experiments. It was obvious, thereafter, that the marbling-effect of the kidneys had to be documented photographically. Therefore, a series of photographs of the entire ventral kidney surface were taken after each CM-injection.

The Bavarian Institutional Animal Care and Use Committee approved the study protocol for the experiments performed in this study (AZ.: 54–2532.1–31/13). All procedures were carried out in accordance with the guidelines and regulations of the Society for Laboratory Animal Science.

### Iodinated contrast media

Two standard CM were applied (Table 3). Iodixanol (320 mg Iodine/ml), GE Healthcare, Munich, Germany, and Iopromide (370 mg Iodine/ml), Bayer/Schering, Berlin, Germany.

**Table 3:** Iodine concentration and osmolality of both iodinated contrast media.

Contrast media	Iodine concentration [mg/ml]	Osmolality [mOsmol/kg H_2_O]
Iodixanol (Visipaque™)	320	290
Iopromide (Ultravist™)	370	770

### Animals

The study was performed on German Landrace pigs with a body weight between 30–35 kg, aged approximately 3 months. There were no significant age or body weight differences between group I and group II. Groups with a maximum of 5 pigs were kept under well-controlled conditions (temperature 15–24 °C; rel. humidity 55 ± 10%; light/dark rhythm 12/12 hours) following the guidelines of the European Societies of Laboratory Animal Sciences. During the evening before the examination, the animals fasted but had access to water *ad libitum*.

### Anaesthesia

An experienced anaesthetist started anaesthesia of the animals with an intramuscular dose of ketamine (600–1000 mg), atropine (0.5–0.8 mg) and azaperone (40–160 mg). Up to 20 minutes later a 20 G catheter was placed in the vein of an ear, enabling additional doses of ketamine or atropine to be administered intravenously as needed. After transfer to the operating theater, an endotracheal tube with a 6.0–6.5 tubus was positioned and 50 mg of rocuronium, 0.5 mg of fentanyl and propofol were given intravenously.

NaCl-solution (0.9%) was infused continuously. Parameters of artificial respiration were as follows: tidal volume 4–4.5 l/min, respiration frequency 16–19/min, oxygen saturation 50%, resulting in an arterial pO_2_ of 130–150 mm Hg, an arterial pCO_2_ of 40–45 mmHg and an arterial pH of 7.4.

### Kidney perfusion measurements using contrast-enhanced ultrasound (CEUS)

**Figure 6:**
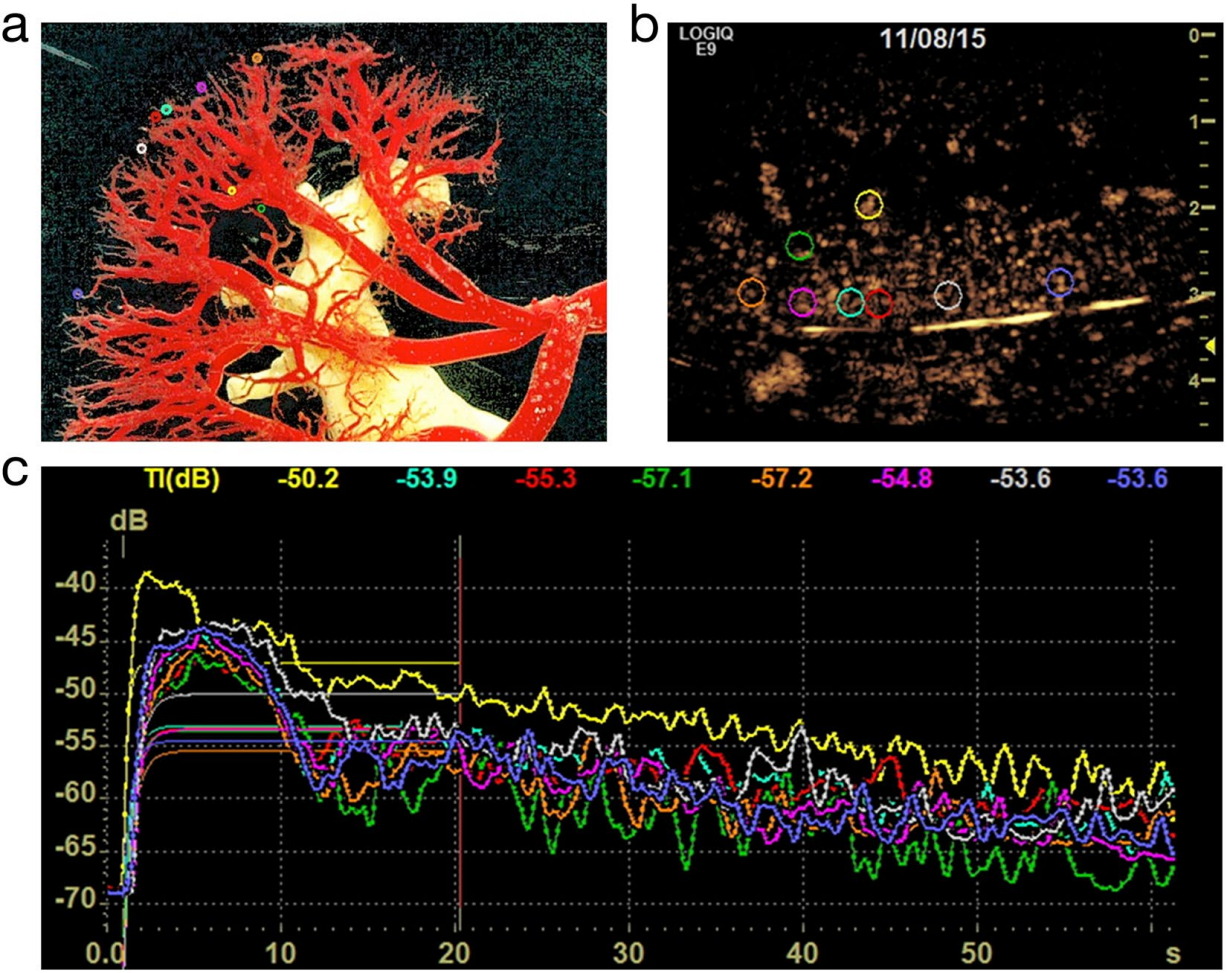
Mean times to peak-intensity in the eight regions of interest (ROI) selected in different kidney regions following the arterial blood vessel hierarchy. (**a**) Position of the ROIs marked in a corrosion cast of the kidney vessels (with permission from the Ruprecht-Karls-University Heidelberg, Institute of Anatomy and Cell Biology, S. Doll). (**b**) Position of the ROIs over an US image in the early phase after injection of US contrast medium. (**c**) Time-intensity curves in the different ROIs over 60 minutes after application of a bolus of US contrast medium into the suprarenal aorta and without application of iodinated CM.

All ultrasound examinations were performed using a state-of-the-art ultrasound machine (LOGIQE9, GE) and high-resolution matrix probes (6–15 MHz, 6–9 MHz, LOGIQ E9, GE, Milwaukee, USA) by an experienced examiner. The ultrasound examination was performed following the renal artery to the cortex using a high-resolution linear probe. The CEUS technology using sulphur-hexafluoride microbubbles (SonoVue™, Bracco, Konstanz, Germany) as contrast medium enabled the assessment of blood flow of internal organs^32^, allowing the *in vivo* examination of the perfusion of renal blood vessels down to the capillary level^33^. Figure 6 shows an example of how kidney perfusion was assessed. After application of microbubbles in the aorta, TTP was measured in different regions of interest (ROI, diameter 5 mm) for up to one minute^34,35^. Figure 6a displays the placement of the ROIs (coloured circles) over a vascular corrosion cast of the kidney and Fig. 6b over an image with microbubbles in renal vessels in the early phase after injection. Figure 6c gives an example of the changes in the amplitudes of the microbubble-dependent intensities during one minute of data sampling immediately after completion of the surgical procedure, without application of a CM.

Microbubble-generated intensity within ROIs was automatically assessed by calculating the mean grey values of all pixels in ROIs in comparison to the mean grey values outside the ROIs^35^. The results were displayed as Time-Intensity-Curves allowing the calculation of the TTP^35^. All ROIs had the same size and were adapted to the size of the first a. arcuata brightly demarcated by CEUS. The ROIs were placed from the a. arcuata to the superficial cortical capillaries, as shown in Fig. 6.

### Blood volume measurement based on CEUS

A computerised offline analysis was performed with perfusion software (Qontrast^®^, Bracco Imaging, Milano, Italy) by the principal investigator who conducted all examinations. A full-map parametric analysis of perfusion within a selected set of frames in a specific ROI^20,36^ can be performed. The loop of images is automatically processed after the tissue region and perfusion period have been defined; translational movements of the selected area can also be corrected. The area is then automatically aligned over all frames, and perfusion is analysed for points that continuously identify the moving tissue. Signal brightness is analysed separately and the optimal fitting curve is evaluated for each point.

In all pigs, a fixed period of 60 seconds after the first appearance of CM was used to create color-coded maps of perfusion parameters to facilitate the interpretation of data. This fixed time period allowed a comparable evaluation of perfusion parameters across all pigs. Time-intensity curves were calculated in 2 ROIs (ROI 1: the complete kidney; ROI 2: the area close to the cortex) and then the differences in the regional blood volume between the baseline values and the values after the tenth bolus of CM were calculated.

### CEUS database assessment of blood vessel diameters

ROIs were placed on three aa. arcuatae randomly selected in the CEUS images of every kidney after CM-bolus 1, bolus 5 and bolus10 and vessel diameters were measured using scale bars offered by the software.

### Radiography

To assess the homogeneity of CM distribution or a possible regional retention of CM, radiography using a SIEMENS Mammomat Nova 3000 was performed 15 minutes after explantation. X-ray images of the post mortem residual CM distribution in the kidneys after repeated CM applications *in vivo* were produced. The images were interpreted by an experienced radiologist, who was blind to the type of contrast agent applied.

### Statistics

With a difference of 10.8 cm/s for the blood flow velocity in the renal artery between both CM and a standard deviation of 5.0 cm/s (estimated according to Mockel M. *et al*.^37^), an α = 0.01, and a sample size of n = 8 pigs per group, a power of 88.8% was calculated.

Data distributions were described by arithmetic means and standard deviations. The testing statistic is performed using analysis of variance for repeated measurements with the factor “iodinated contrast media”. Probability of error (p) values < 0.05 were regarded as statistically significant.

### Data availability

The datasets generated and analysed during the current study are available from the corresponding author upon request.

Figure 6a is provided with permission from the Ruprecht-Karls-University Heidelberg, Institute of Anatomy and Cell Biology, S. Doll (official permission attached to the manuscript file).

### Disclosure

The study was supported by a grant from GE Healthcare.
